# Supplementary material for: Canine detection of explosives under adverse environmental conditions with and without acclimation training
Source: PLoS One. 2024 Feb 21;19(2):e0297538. doi: 10.1371/journal.pone.0297538 (PMC10881013; doi:10.1371/journal.pone.0297538)
Supplement: Attachment — Submitted filename: PONE-D-23-31666.docx [file pone.0297538.s007.docx]

**PLOS ONE**

**Canine detection of explosive odorants in extreme environmental conditions and development of an acclimatization plan to improve sensitivity**

--Manuscript Draft--

| **Manuscript Number:** | PONE-D-23-31666 |
| --- | --- |
| **Article Type:** | Research Article |
| **Full Title:** | Canine detection of explosive odorants in extreme environmental conditions and development of an acclimatization plan to improve sensitivity |
| **Short Title:** | Canine detection in extreme environmental conditions and development of an acclimatization plan. |
| **Corresponding Author:** | Sarah A. Kane  Texas Technical University: Texas Tech University Lubbock, TX UNITED STATES |
| **Keywords:** | Canine, canine olfaction; environment; threshold limit; detection; explosives detection canines |
| **Abstract:** | Canines are one of the best biological detectors of energetic materials available; however, canine detection of explosives does have limitations. The objectives of this study were: 1) determine how canine detection limits vary when both the canine and odorant are held in varying temperature and humidity conditions (canine and odor interactive effects); and 2) determine if an acclimatization plan can improve detection limits in an adverse environmental condition. Eight working line canines were trained to detect four energetics: prill ammonium nitrate (AN), Composition 4 (C4), trinitrotoluene (TNT) and double base smokeless powder (SP). In Experiment 1, canines completed a 3-alternative forced choice 3-down-1-up staircase threshold assessment in five environmental conditions: 40°C and 70% relative humidity (RH), 40°C and 40% RH, 0°C and 90% RH, 0°C and 50% RH and 21°C and 50% RH. Canines showed a 3.5- fold detection limit increase (poorer detection) for C4 in 40°C and 70% RH compared to their detection limit at 21°C and 50% RH. In Experiment 2, the eight canines were split into two groups (n=4), control and acclimation groups. The control group completed the threshold assessment for C4 at 21°C and 50% RH each day for 20 days. The acclimation group completed the same assessment daily starting at 21°C and 50% RH but temperature and RH were incremented daily over the course of 6 days to the 40°C and 70% RH condition. After the initial six days, the acclimation group completed daily assessments at 40°C and 70% RH condition for the remainder of the experiment. Detection limits for C4 for all dogs were tested in 40°C and 70% RH on day 11 and day 22. The acclimatization plan improved detection limits in the 40°C and 70% RH condition for C4 compared to the non-acclimated group. In this set of experiments, canine detection limits for four explosive odorants were found to vary based on environmental condition and were mostly driven by impacts on the canine rather than odor availability. The acclimatization plan did result in lower detection limits (i.e., increased performance). |
| **Order of Authors:** | Sarah A. Kane |
|  | Lauren S. Fernandez |
|  | Dillon E. Huff, PhD |
|  | Paola A. Prada-Tiedemann, PhD |
|  | Nathaniel J. Hall, PhD |
| **Additional Information:** |  |
| **Question** | **Response** |
| **Financial Disclosure**  Enter a financial disclosure statement that describes the sources of funding for the | Yes |

*Powered by Editorial Manager® and ProduXion Manager® from Aries Systems Corporation*

| work included in this submission. Review the [submission guidelines](http://journals.plos.org/plosone/s/submission-guidelines#loc-financial-disclosure-statement) for detailed requirements. View published research articles from [*PLOS ONE*](https://journals.plos.org/plosone/) for specific examples.  This statement is required for submission and **will appear in the published article** if the submission is accepted. Please make sure it is accurate. |  |
| --- | --- |
| **Funded studies**  Enter a statement with the following details:   - Initials of the authors who received each award - Grant numbers awarded to each author - The full name of each funder - URL of each funder website - Did the sponsors or funders play any role in the study design, data collection and analysis, decision to publish, or preparation of the manuscript? |  |
| Did you receive funding for this work? |  |
| Please add funding details.  as follow-up to "**Financial Disclosure**  Enter a financial disclosure statement that describes the sources of funding for the work included in this submission. Review the [submission guidelines](http://journals.plos.org/plosone/s/submission-guidelines#loc-financial-disclosure-statement) for detailed requirements. View published research articles from [*PLOS ONE*](https://journals.plos.org/plosone/) for specific examples.  This statement is required for submission and **will appear in the published article** if the submission is accepted. Please make sure it is accurate. | This research was made possible through funding provided by the DoD Army Research Office under Contract No. W911NF2120124. The views expressed in this manuscript are those of the author and do not necessarily reflect the official policy or position of the Department of Defense, nor the U.S. Government. [https://www.arl.army.mil/who-we-are/aro/](http://www.arl.army.mil/who-we-are/aro/)  SAK’s work was supported by the National Science Foundation Graduate Research Fellowship Program (DGE 2140745). Any opinions, findings, conclusions or recommendations expressed in this work are those of the author(s) and do not necessarily reflect the views of the National Science [Foundation.https://www.nsf.gov/](http://www.nsf.gov/) |

*Powered by Editorial Manager® and ProduXion Manager® from Aries Systems Corporation*

| **Funded studies**  Enter a statement with the following details:   - Initials of the authors who received each award - Grant numbers awarded to each author - The full name of each funder - URL of each funder website - Did the sponsors or funders play any role in the study design, data collection and analysis, decision to publish, or preparation of the manuscript? |  |
| --- | --- |
| Did you receive funding for this work?" |  |
| Please select the country of your main research funder (please select carefully as in some cases this is used in fee calculation).  as follow-up to "**Financial Disclosure**  Enter a financial disclosure statement that describes the sources of funding for the work included in this submission. Review the [submission guidelines](http://journals.plos.org/plosone/s/submission-guidelines#loc-financial-disclosure-statement) for detailed requirements. View published research articles from [*PLOS ONE*](https://journals.plos.org/plosone/) for specific examples.  This statement is required for submission and **will appear in the published article** if the submission is accepted. Please make sure it is accurate. | UNITED STATES - US |
| **Funded studies**  Enter a statement with the following details:   - Initials of the authors who received each award - Grant numbers awarded to each author - The full name of each funder - URL of each funder website - Did the sponsors or funders play any role in the study design, data collection and analysis, decision to publish, or preparation of the manuscript? |  |
| Did you receive funding for this work?" |  |
| **Competing Interests** | The authors have declared that no competing interests exist. |

*Powered by Editorial Manager® and ProduXion Manager® from Aries Systems Corporation*

| Use the instructions below to enter a competing interest statement for this submission. On behalf of all authors, disclose any [competing interests](http://journals.plos.org/plosone/s/competing-interests) that could be perceived to bias this work—acknowledging all financial support and any other relevant financial or non- financial competing interests.  This statement is required for submission and **will appear in the published article** if the submission is accepted. Please make sure it is accurate and that any funding sources listed in your Funding Information later in the submission form are also declared in your Financial Disclosure statement.  View published research articles from  [*PLOS ONE*](http://journals.plos.org/plosone/) for specific examples. |  |
| --- | --- |
| **NO authors have competing interests**  Enter: *The authors have declared that no competing interests exist.*  **Authors with competing interests**  Enter competing interest details beginning with this statement:  *I have read the journal's policy and the authors of this manuscript have the following competing interests: [insert competing interests here]* |  |
| * typeset |  |
| **Ethics Statement**  Enter an ethics statement for this submission. This statement is required if the study involved:   - Human participants - Human specimens or tissue - Vertebrate animals or cephalopods - Vertebrate embryos or tissues | This project was conducted at Texas Tech University and was overseen by the Institutional Animal Care and Use Committee (Protocol #21051-07) and approved by the US Army Medical Research and Development Command Animal Care and Use Office (#78018-ST-H.e001). In addition to their participation in this project, all the dogs had additional enrichment activities every day and received food enrichment in their kennels. Throughout all testing, all dogs always had access to water. During testing if canines refused to search for 5 trials in a row or lay down and did not get up when called, testing was stopped, and they were immediately removed from the environment. This termination criterion was used to ensure participant safety; this criterion never occurred under standard conditions and was indicative of lack of |

*Powered by Editorial Manager® and ProduXion Manager® from Aries Systems Corporation*

| - Field research   Write "N/A" if the submission does not require an ethics statement.  General guidance is provided below. Consult the [submission guidelines](http://journals.plos.org/plosone/s/submission-guidelines#loc-guidelines-for-specific-study-types) for detailed instructions. **Make sure that all information entered here is included in the Methods section of the manuscript.** | performance. |
| --- | --- |

*Powered by Editorial Manager® and ProduXion Manager® from Aries Systems Corporation*

| **Format for specific study types**  **Human Subject Research (involving human participants and/or tissue)**   - Give the name of the institutional review board or ethics committee that approved the study - Include the approval number and/or a statement indicating approval of this research - Indicate the form of consent obtained (written/oral) or the reason that consent was not obtained (e.g. the data were analyzed anonymously)   **Animal Research (involving vertebrate animals, embryos or tissues)**   - Provide the name of the Institutional Animal Care and Use Committee (IACUC) or other relevant ethics board that reviewed the study protocol, and indicate whether they approved this research or granted a formal waiver of ethical approval - Include an approval number if one was obtained - If the study involved *non-human primates*, add *additional details* about animal welfare and steps taken to ameliorate suffering - If anesthesia, euthanasia, or any kind of animal sacrifice is part of the study, include briefly which substances and/or methods were applied   **Field Research**  Include the following details if this study involves the collection of plant, animal, or other materials from a natural setting:   - Field permit number - Name of the institution or relevant body that granted permission |  |
| --- | --- |
|  |  |
| **Data Availability**  Authors are required to make all data underlying the findings described fully available, without restriction, and from the time of publication. PLOS allows rare exceptions to address legal and ethical concerns. See the [PLOS Data Policy](http://journals.plos.org/plosone/s/data-availability) and [FAQ](http://journals.plos.org/plosone/s/data-availability#loc-faqs-for-data-policy) for detailed information. | Yes - all data are fully available without restriction |

*Powered by Editorial Manager® and ProduXion Manager® from Aries Systems Corporation*

| A Data Availability Statement describing where the data can be found is required at submission. Your answers to this question constitute the Data Availability Statement and **will be published in the article**, if accepted. |  |
| --- | --- |
| **Important:** Stating ‘data available on request from the author’ is not sufficient. If your data are only available upon request, select ‘No’ for the first question and explain your exceptional situation in the text box. |  |
| Do the authors confirm that all data underlying the findings described in their manuscript are fully available without restriction? |  |
| **Describe where the data may be found in full sentences. If you are copying our sample text, replace any instances of XXX with the appropriate details.**   - If the data are **held or will be held in a public repository**, include URLs, accession numbers or DOIs. If this information will only be available after acceptance, indicate this by ticking the box below. For example: *All XXX files are available from the XXX database (accession number(s) XXX, XXX.*). - If the data are all contained **within the manuscript and/or Supporting Information files**, enter the following: *All relevant data are within the manuscript and its Supporting Information files.* - If neither of these applies but you are able to provide **details of access elsewhere**, with or without limitations, please do so. For example:   *Data cannot be shared publicly because of [XXX]. Data are available from the XXX Institutional Data Access / Ethics Committee (contact via XXX) for researchers who meet the criteria for access to confidential data.*  *The data underlying the results presented in the study are available from (include the name of the third party* | All relevant data are within the manuscript and its Supporting Information files. |

*Powered by Editorial Manager® and ProduXion Manager® from Aries Systems Corporation*

| *and contact information or URL).*   - This text is appropriate if the data are owned by a third party and authors do not have permission to share the data.   * typeset |  |
| --- | --- |
| Additional data availability information: |  |

*Powered by Editorial Manager® and ProduXion Manager® from Aries Systems Corporation*

Manuscript	[Click here to](https://www.editorialmanager.com/pone/download.aspx?id=34321637&guid=b27f15eb-df37-459e-8159-27009e8a8ab7&scheme=1)

[access/download;Manuscript;Kane_PLOS_ONE_submission.doc](https://www.editorialmanager.com/pone/download.aspx?id=34321637&guid=b27f15eb-df37-459e-8159-27009e8a8ab7&scheme=1)

1	Canine detection of explosive odorants in

2	extreme environmental conditions and

3	development of an acclimatization plan to

4	improve sensitivity

5

6 Sarah A. Kane^1^, Lauren S. Fernandez^1^, Dr. Dillon E. Huff^2^, Dr. Paola A. Prada-Tiedemann^2^, Dr.
7 Nathaniel J. Hall^1^ 8
9 ^1^Canine Olfaction Research and Education Lab, Davis College of Animal and Food Science, Texas
10 Tech University, 1308 Indiana Ave, Lubbock, TX.
11 ^2^ Forensic Analytical Chemistry and Odor Profiling Laboratory, Department of Environmental
12 Toxicology at Texas Tech University, 1207 S Gilbert Dr, Lubbock, TX.

# 13 Funding statement

14

15 This research was made possible through funding provided by the DoD Army Research
16 Office under Contract No. W911NF2120124. The views expressed in this manuscript are those
17 of the author and do not necessarily reflect the official policy or position of the Department of
18 Defense, nor the U.S. Government.
19 SAK’s work was supported by the National Science Foundation Graduate Research
20 Fellowship Program (DGE 2140745). Any opinions, findings, conclusions or recommendations
21 expressed in this work are those of the author(s) and do not necessarily reflect the views of the
22 National Science Foundation. 23

# 24 Abstract

25 Canines are one of the best biological detectors of energetic materials available; however,
26 canine detection of explosives does have limitations. The objectives of this study were: 1)
27 determine how canine detection limits vary when both the canine and odorant are held in varying
28 temperature and humidity conditions (canine and odor interactive effects); and 2) determine if an
29 acclimatization plan can improve detection limits in an adverse environmental condition. Eight
30 working line canines were trained to detect four energetics: prill ammonium nitrate (AN),
31 Composition 4 (C4), trinitrotoluene (TNT) and double base smokeless powder (SP). In
32 Experiment 1, canines completed a 3-alternative forced choice 3-down-1-up staircase threshold
33 assessment in five environmental conditions: 40°C and 70% relative humidity (RH), 40°C and
34 40% RH, 0°C and 90% RH, 0°C and 50% RH and 21°C and 50% RH. Canines showed a 3.5-
35 fold detection limit increase (poorer detection) for C4 in 40°C and 70% RH compared to their
36 detection limit at 21°C and 50% RH. In Experiment 2, the eight canines were split into two
37 groups (n=4), control and acclimation groups. The control group completed the threshold
38 assessment for C4 at 21°C and 50% RH each day for 20 days. The acclimation group completed
39 the same assessment daily starting at 21°C and 50% RH but temperature and RH were
40 incremented daily over the course of 6 days to the 40°C and 70% RH condition. After the initial
41 six days, the acclimation group completed daily assessments at 40°C and 70% RH condition for
42 the remainder of the experiment. Detection limits for C4 for all dogs were tested in 40°C and
43 70% RH on day 11 and day 22. The acclimatization plan improved detection limits in the 40°C
44 and 70% RH condition for C4 compared to the non-acclimated group. In this set of experiments,
45 canine detection limits for four explosive odorants were found to vary based on environmental
46 condition and were mostly driven by impacts on the canine rather than odor availability. The
47 acclimatization plan did result in lower detection limits (i.e., increased performance).

48

49

# 50 Introduction

51 Explosive detection canines (EDCs) are a critical component in public and military
52 safety. Currently, these specially trained canines are one of the best detectors for explosives
53 because of their mobility, and capacity to deploy in a variety of environments (1). EDCs are able
54 to search environments which other detection technologies cannot access (1). In addition, EDCs
55 can be deployed in a variety of environments, both indoor and outdoor (2). Farr et al. (2021)
56 surveyed EDC handlers who reported they were regularly deployed to search in climates to
57 which they had limited acclimation prior to searching.
58 Although the versatility of EDCs is a benefit, further research is needed to understand
59 how temperature and humidity in particular can affect their detection abilities (3). Because EDCs
60 can be rapidly deployed to harsh environments, it is crucial to know how these environmental
61 changes can impact their detection limits. In addition, such research could provide a useful
62 resource for handlers on best practices for working EDCs in adverse conditions.
63 Much of what we know about environmental effects on detection limits does not come
64 from EDC literature, but from conservation detection dogs. In one such study, relative humidity
65 and air temperature were not correlated with detection rates of the desert tortoise (4). This lack of
66 a result could be procedural as canine teams were rested once the dog showed behavioral signs of
67 heat stress, or their rectal temperature was greater than 40°C. Because dogs were only worked
68 when they were in optimal physiological conditions, fatigue or stress caused by the environment
69 did not impact their detection rates. This study did note that canine fatigue was a limiting factor
70 in duration of search (4). It was unclear if that fatigue was due to the physical demands of the
71 task, the environmental conditions in which the canines worked, or a combination of both.
72 Another study, however, found that temperature did affect detection rates; higher air
73 temperatures and higher windspeeds led to greater detection distances (better detection) (5).
74 Unfortunately, due to the lack of climactic variation in this study, further conclusions on the
75 effect of climate (specifically relative humidity) on detection were limited.
76 Gazit and Terkel (2003) studied how physiological factors, specifically exercise induced
77 fatigue, affected canine detection of explosives. Gazit and Terkel (2003) compared canines’
78 ability to detect C4 in indoor and outdoor search areas under two conditions: after rest and after
79 strenuous physical activity. They found that canines were always able to detect the explosive
80 regardless of condition, in the indoor search, however after strenuous activity, canines missed
81 explosives in their outdoor search. Search time was also increased, both indoor and outdoor, after
82 exercise. Gazit and Terkel (2003) suggested that the decreased ability to detect C4 after activity
83 was because of changes in the canines’ sniffing to panting ratio. In order to maintain homeostasis
84 during and after exercising canines need to pant to cool down via evaporative cooling (6). Gazit
85 and Terkel posited that as panting increased, sniffing deceased, and thus detection of C4
86 decreased. Canines did show acclimatization throughout the study to detect C4 after exercise (7).
87 Gazit and Terkel (2003) provide some of the only evidence exploring canine detection of
88 explosives and physiological state.
89 Temperature and humidity can also change physiological state of the working canine. Past
90 research in our laboratory has studied how canine physiological factors alone affect detection
91 limits of narcotic signatures and explosives (Brustkern et al., 2023; Fernandez et al., in review).
92 Fernandez et al. (in review) worked canines through a go/no-go threshold assessment with four
93 different explosive targets. The canines worked in an environmentally controlled chamber;
94 however, the odorant was kept outside of the research area in a temperature-controlled water
95 bath. The purpose of this study was to determine how canine physiological factors alone affected
96 detection limits for explosives. This study found that canines had lowest detection limits in
97 standard (22°C and 50% RH) conditions compared to environments with higher or lower
98 temperatures and RH levels. A similar study which used methyl benzoate as the target material,
99 also kept the odorant in a consistent environment while the dog worked in a variety of
100 temperature and humidity conditions (8). This study found that canines had poorer detection in
101 high temperature conditions compared to standard, but did not see a performance decrement in
102 low temperature conditions. This study also suggested in the first set of threshold detection test
103 results that canines had poorer detection immediately upon entering the harsh environment from
104 a controlled condition, i.e. there was a “startup” decrement (8).
105 Odor availability is also affected by the environment (1). In high temperature and relative
106 humidity conditions more volatiles of an explosive enter the headspace. This should allow for
107 greater availability for canine detection. Conversely, colder conditions decrease volatiles in the
108 headspace and lead to decreased odor availability (Huff, et al., in prep). Both odor and canine
109 physiological state fluctuate with environmental conditions, and these interactions determine
110 detection limits. Huff et al. (in prep) studied how odors behave in a variety of temperature and
111 relative humidity conditions using SPME and GC-MS techniques. When sampling occurred in
112 hot conditions, volatiles in the headspace increased. In colder conditions, the volatiles in the
113 headspace decreased. No study, that these authors are aware of, has yet examined how odor
114 availability and canine physiological variability interact when in adverse environmental
115 conditions together.
116 Both physiological factors on the canine and odor availability affect detection limits,
117 however due to lack of research in this area, the relationship between these factors is unclear. It
118 is possible that any physiological challenges dogs experience in high temperatures could be
119 mitigated by increased odor availability in these temperatures. In cold conditions odor is less
120 available, but this effect may be lessened if the dogs are not under physiological stress (i.e.
121 panting) when working in this condition.
122 It is evident in the literature that there is a lack of conclusive support on how climate, odor
123 and canine detection, interact. The objective of the first experiment of this study was to
124 determine how detection limits of canines are affected by environmental condition when both
125 odorant and canine are held in the same environmental conditions. The second experiment
126 objective was to develop an acclimatization plan to improve detection in adverse conditions.

# 127 Experiment 1: Characterizing canine detection sensitivity in varying

128 environments

### 129 Materials and Methods

130 Participants
131 Eight dogs (6M, 2F; 4 Labrador retrievers, 4 German Short-Haired Pointers), former
132 candidates of a government detection program, participated in this project. The dogs had various
133 amounts of prior training with explosives.
134 Animal welfare considerations
135 This project was conducted at Texas Tech University and was overseen by the
136 Institutional Animal Care and Use Committee (Protocol #21051-07) and approved by the US
137 Army Medical Research and Development Command Animal Care and Use Office (#78018-ST-
138 138
139 139
140 140
141 141
142 142
143 143
144 144
145 145
146 146
147 147
148 148
149 149
150 150
151 151
152 152
153 153
154 154
155 155
156 156

H.e001). In addition to their participation in this project, all the dogs had additional enrichment activities every day and received food enrichment in their kennels. Throughout all testing, all dogs always had access to water. During testing if canines refused to search for 5 trials in a row or lay down and did not get up when called, testing was stopped, and they were immediately removed from the environment. This termination criterion was used to ensure participant safety; this criterion never occurred under standard conditions and was indicative of lack of performance.

Odorants

Four odorants were used in this project; C4, double-based smokeless powder, ammonium nitrate prill (explosives grade), and TNT (see Table 1 for amounts and brands). Use of all energetic materials was approved by the Institutional Laboratory Safety Committee on Energetic Materials (Protocol ILSC#2110E1). There were no distractor odorants, all other valves of the olfactometer (described below) were connected to empty borosilicate glass vials matching the type used to contain the target odorants.

*Table 1: Explosive material used as target odorants in Experiment 1. Vial diameter is specified because surface area impacts odor availability. Explosives held in a vial with a larger diameter had a higher odor availability than if they had been held in a vial with a smaller diameter. Larger diameter vials were used when odors were difficult to detect even at the initial standard condition.*

Odor	Mass per vial (g)	Brand of energetic material

Vial diameter (cm)

Double-base smokeless powder (SP)

Ammonium nitrate (AN)

Trinitrotoluene (TNT)

1.039g	Hodgdon H335	2.54

20.422g	Omni Explosives	5.08

20.6	Omni Explosives	5.08

C4	4.23g	Omni Explosives	2.54

157 157
158 Data collection equipment
159 Three Bluetooth enabled olfactometers were used in this project, which were a
160 modification of our prior automated line up equipment (see Aviles-Rosa et al., 2021). The odor
161 generation design was identical, but instead of having a horizonal panel of odor ports, the odor
162 port was integrated into the olfactometer box itself. Additionally, the programing was modified
163 to allow Bluetooth control of the olfactometers.
164 Each olfactometer was an independent replicate containing the same odor delivery
165 system and odorants. Each olfactometer had six vials: one vial contained the target odorant (C4,
166 TNT, AN, or SP) while the remaining five vials were blank controls. These control vials were
167 cleaned and prepared in a manner identical to the target vial.
168 Odor was generated from a 4L/minute oil-less air pump. Air from the pump first passed
169 through a charcoal filter to scrub the air of odorants. The air was then split to an odor line and a
170 dilution line via a T-connector. The odor line was regulated by a 0-1 lpm rotameter and the
171 dilution line was regulated by a 0-4 lpm rotameter. From that regulating rotameter, the odor line
172 passed to a manifold with one-way valves. Upon activation of a valve, the odor line flow was
173 passed into a vial containing the odorant (or a clean distractor vial). This displaced the headspace
174 of the vial through a one-way check valve into a PTFE mixing manifold where it mixed with
175 clean air from the regulated clean air. Air from this manifold flowed to the port for the dog to
176 sniff.
177 The olfactometer was controlled by a custom program
178 (https://github.com/njhall1/CanineOlfactometer). Odor dilutions were controlled by manual
179 manipulation of the flowmeters controlling the odor line and dilution line. Thus, dilution was
180 created as an air dilution by changing the ratio of air from the odor and dilution lines. The
181 flowmeters allowed a dilution from 80 to 3% (see Table 2). For threshold testing, a series of
182 dilutions was designed: 80%, 50%, 25%, 12% and 3%. The flow rate, or the amount of odor
183 being pushed into the port was consistent for each concentration, except for 80%. To reach to the
184 80% concentration, the flow rate had to be lower, however all three olfactometers delivered the
185 same flow rate.
186 186
187 *Table 2: Odor concentration changes for the 3 down 1 up staircase threshold procedure*

|  | Concentration (%  odor line to clean air line) | Step number | Odor line flow rate (cc/min) | Clean line flow rate (L/min) |
| --- | --- | --- | --- | --- |
|  | 80% | 1 | 1000 | 0.25 |
|  | 50% | 2 | 1000 | 1.0 |
|  | 25% | 3 | 750 | 2.25 |
|  | 12% | 4 | 360 | 2.65 |
|  | 3% | 5 | 90 | 2.91 |
| 188 |  |  |  |  |

189 Each olfactometer contained an infrared sensor beam pair that measured all canine entries
190 into the odor ports. These IRs also recorded when the beam was broken so that duration between
191 nose pokes in each box could be calculated. We also calculated latency, or the duration from start
192 of the trial to the time the dog began search (as indicated by a nose poke breaking the IR beam).
193 This study utilized an alternative forced choice test design, in which the target odor was
194 always present in one of the three olfactometers. The other two olfactometers were pushing the
195 same volume of air from one of the five control (empty) vials.
196 196
197 Temperature sensor
198 Each dog had a subcutaneous microchip over their shoulder which read their
199 subcutaneous body temperature. At the start of every testing session, approximately every 5
200 minutes during the session, at the end of the session and 5-minutes post session, an experimenter
201 scanned the 134.2 kHz ISO microchip with a HomeAgain™ Universal Worldscan™ Reader Plus
202 (by MERCK animal health) to record the subcutaneous temperature of the dog (9).
203 Environmental conditions
204 There were five temperature and humidity conditions used in this experiment (see Table
205 3). Experimenters allowed the standard, low-temperature high humidity, high temperature low
206 humidity and high temperature high humidity to vary by 3°C and 5% relative humidity (RH)
207 from the stated condition to allow for natural fluctuations as airflow changed when the door of
208 the environmental chamber was opened, or the AC unit began a thaw cycle. The low temperature
209 low humidity condition was allowed to vary by 3°C and 10% RH due to ambient humidity
210 levels.
211 Dogs were tested once under each condition for each target odor. All dogs were first
212 tested with each odor under standard conditions. Four dogs were randomly assigned to one of
213 two testing orders (A or B; see
214 Table 4 for order). Each odor was tested to completion before moving to the next to
215 reduce the potential of olfactometer contamination by switching odors multiple times per day.
216 Threshold limits were determined first for C4 then SP, AN, and last TNT.
217 *Table 3: Temperature and humidity conditions for each of the 5 environmental conditions and the allowed for variation in the*
218 *environmental condition set point.*

|  | **Condition** | **Temperature (°C)** | **Relative humidity (%**  **RH)** |
| --- | --- | --- | --- |
|  | Standard (STD) | 21 ± 3 | 50 ± 5 |
|  | High temperature high humidity (HTHH) high temperature low humidity (HTLH) Low temperature high humidity (LTHH) Low temperature low humidity (LTLH) | 40 ± 3  40 ± 3  0 ± 3  0 ± 3 | 70 ± 5  40 ± 5  90 ± 5  50 ± 10 |
| 219 |  |  |  |

220

221

222

223

*Table 4: Indicates the orders each group of dogs worked through the conditions for each target odorant. Blocking of testing order was done to help limit order effects. STD=standard, HTHH= high temperature high humidity, HTLH=high temperature low humidity, LTHH= Low temperature high humidity, LTLH= low temperature low humidity. Odors ordered in the table as they were presented to the canines, top to bottom. Groups were consistent throughout experiment 1.*

|  | **Groups (n=4)** | **Odor** | **Cond 1** | **Cond 2** | **Cond 3** | **Cond 4** | **Cond 5** |
| --- | --- | --- | --- | --- | --- | --- | --- |
|  | Group A | C4 | STD | LTHH | HTHH | HTLH | LTLH |
|  | Group B | C4 | STD | HTLH | LTLH | LTHH | HTHH |
|  | Group A | SP | STD | HTLH | LTHH | LTLH | HTHH |
|  | Group B | SP | STD | LTHH | HTLH | HTHH | LTLH |
|  | Group A | AN | STD | HTHH | LTLH | LTHH | HTLH |
|  | Group B | AN | STD | LTHH | HTHH | LTLH | HTLH |
|  | Group A | TNT | STD | LTHH | HTHH | HTLH | LTLH |
|  | Group B | TNT | STD | HTHH | HTLH | LTLH | LTHH |
| 224 |  |  |  |  |  |  |  |

225 Experimental Set up
226 Research was conducted in a 3.6 x 3.4 m environmental chamber. The room was
227 equipped with a heater, AC unit, dehumidifiers (hOmeLabs and ALORAIR), humidifiers
228 (AILINKE), a fan and a mister system (homenote Misting Cooling System). The misters hung
229 from the ceiling to help the room reach humidity conditions. The room also contained a
230 SensorPush HT.w wireless thermometer/hygrometer sensor which measured the humidity and
231 temperature in the room (rated accuracy: ± 2% RH and ± 0.2°C). Three Bluetooth enabled
232 olfactometer boxes were arranged as a triangle in the room and presented all odors for canines
233 (Figure 1).
234 234
235 *Figure 1: Environmental chamber room set up (overhead view). The humidifier and dehumidifier number varied based on*
236 *conditions requirements. The Bluetooth enabled olfactometer boxes (3) or BLE olfactometers are pictured.*
237 Odor availability analysis
238 A solid phase microextraction headspace sampling was performed on all target explosive
239 odorants while being exposed to each extreme condition inside the environmental chamber. This
240 extraction mode aimed to understand how the chemical odor profile remained during an “active
241 operational” environmental effect. Each environmental condition and target odorant combination
242 included a total of ten replicate samples.
243 All chromatographic separations utilized an Agilent 7890A GC Series GC system,
244 equipped with front and rear split/splitless injection ports, fitted with an HP5 30 m, 0.25 mm
245 internal diameter capillary column. Helium was the carrier gas at a flow rate of 1 mL min-1 at an
246 average velocity of 37 cm s-1. The mass spectrometer (MS) operated in electron ionization (EI)
247 full scan mode from 45 to 550 amu, with a 3-min solvent delay. All compounds in the headspace
248 were assigned based on mass spectral matches to the NIST 2017 mass spectral library.
249 Quantification of target VOC concentrations being extracted by the SPME fiber was
250 performed via external calibration using a standard mix of signature VOCs within each explosive
251 class. For the target VOCs of each explosive class, the calibration curve range between 1-100 mg
252 L^-1^ to demonstrate the fiber capacity to extract target analytes at trace concentrations in addition
253 to higher concentrations quantified above 100ppm without losing linearity as observed with the
254 linear dynamic range coefficients of > 0.98. To approximate the amount of VOCs extracted by
255 the SPME fiber, the slope of the line obtained in the calibration curve was used as a response
256 factor for each of the analyzed compounds.
257 257
258 Canine odor detection testing and training
259 All canines had been previously trained to detect explosives in an olfactometer during a
260 previous study (Fernandez, et al., in review). Dogs were trained to detect the target odorants in a
261 3 alternative forced choice test paradigm prior to starting threshold testing. Canines all reached
262 85% accuracy at the 80% concentration across 20 trials for each odor before beginning threshold
263 testing on that odor.
264 All training and testing was conducted under double blind conditions such that the
265 handler was never aware of the location of the olfactometer presenting the target odor. Canine
266 responses (i.e. alerts), were scored automatically using infrared beam pairs. All dogs had a nose
267 hold and freeze alert response for the target odorants. If dogs made a correct response, a tone
268 played, and the handler provided a reinforcer to the dog. If the dog made a false alert or failed to
269 respond to the correct port, an “incorrect” tone played. When the dog was incorrect no reinforcer
270 was given. At the end of the trial, there was a 20s inter-trial interval to purge the olfactometers of
271 any residual odor.
272 Canine odor threshold testing
273 The threshold testing was conducted as an alternative forced choice task with a 3-down 1-
274 up descending staircase procedure. In an alternative forced choice task, the target odorant was
275 present in one of the three olfactometers in every trial. In the 3- down, 1- up threshold test
276 procedure, after three consecutive correct trials the odor concentration was decreased by one
277 dilution step. If the dog made an incorrect response, the odor concentration was increased by one
278 step. A reversal point was defined as anytime the direction of odorant concentration changed.
279 Threshold testing continued until dogs completed seven reversals. Testing also terminated if
280 dogs made three consecutive correct responses at the lowest concentration step (3%).
281 Additionally, testing also terminated if dogs met a welfare criterion by not searching for five
282 consecutive trials or showed signs of elevated heat stress and refused to search for one trial.
283 Elevated heat stress signals consisted of a dog lying down in the testing chamber, excessively
284 panting, and refusing to continue searching for one trial. The criterion of trials not searching was
285 reduced when accompanied by these additional signs of heat stress.
286 286
287 Data Analysis
288 Odor detection threshold was calculated as the geometric mean of all seven reversal
289 points. Threshold was log transformed for all analyses. If dogs’ detection exceeded the
290 maximum dilution capabilities of the olfactometer (3 correct responses at the lowest
291 concentration), the lowest dilution point was used as a reversal point for the remaining reversal
292 points of that session. For example, if a dog reached 3% by reversal 2, the remaining five
293 reversal point concentrations were imputed as 0.03. If a dog terminated a session early due to
294 meeting the welfare criterion, the remaining reversal points were imputed as 80%, indicating
295 failure to complete the task.
296 296
297 To evaluate the effect of environmental conditions on detection limits, a linear mixed
298 effect regression model was fit in which log threshold was predicted by breed, environmental
299 condition, odor and an interaction between environmental condition and odor, with a random
300 effect of individual dog. This model produced a residual plot with some boundary effects, due to
301 the limitations of the highest and lowest concentrations produced by the olfactometers

*Figure 2 Detection limit (log scale) for each odorant in each environmental condition. The figure indicates that the detection limit for smokeless powder (SP) was not reached. The points on the graph are the average log geometric mean thresholds for all dogs in each condition for each odor. The error bars represent the 95% confidence interval for those geometric means.*

302 (maximum concentration was 80% and minimum was 3%). Otherwise, the residual plot showed
303 no patterns and so the model was deemed fit for interpretation. Models were fit in R using the
304 lme4 package (10) and significance interpretation was made using the Anova function from the
305 car package (11) and lsmeans from the emmeans package (12).
306 306

### 307 Results and Discussion

308 The thresholds for each odor in each condition can be seen in Table 5 and Figure 2. The
309 detection limits (log scale) for each odor varied by condition and odor identity, as was seen by
310 Fernandez et al. (in review). For smokeless powder, dogs’ detection limits were lower than the
311 dilutions available by the Bluetooth olfactometers. This was expected based on the detection
312 limits observed by Fernandez et al. (in review).
313 The linear mixed effect model indicated that there was a significant effect of odor

314	(Ꭓ^2^=181 DF=3, P<0.0001), environmental conditions (Ꭓ^2^= 13.03, DF=4, P=0.01) and their

315	interaction (Ꭓ^2^= 26.4, DF= 12, P<0.01) on detection limit. Breed, however, had no impact (Ꭓ^2^=

316	0.22, DF=1, P=0.64).

317 Post hoc tests were conducted comparing each environmental condition to standard
318 conditions for each target odor (see
319 Table 6). Figure 2 shows the average geometric means for all dogs in each condition for
320 each odor and the 95% confidence intervals. Post hoc tests showed that detection limits for C4
321 were higher (poorer) under high temperature high humidity (est=0.47, t=2.7, p=0.03) and high
322 temperature low humidity conditions (est=0.38, t=2.3, p=0.05). Low temperature low humidity
323 and low temperature high humidity conditions did not differ from standard conditions (p>0.05).
324 Smokeless powder thresholds did not differ between environmental conditions (all p>0.05), but
325 this may reflect that dogs’ thresholds was below the limits of dilution of the olfactometer.
326 For AN, poorest detection limits occurred under the hot temperature and low temperature
327 high humidity conditions, however these differences from standard did not reach the statistical
328 significance criterion (all p>0.05). For TNT, dogs showed detection difficulty in all conditions
329 and no environmental condition differed from standard.
330 *Table 5: Air Dilution detection limit (on the log scale) by odor in each condition.*

|  | **Odor** | **Standard** | **High Temp**  **High Humid** | **High Temp**  **Low Humid** | **Low Temp**  **High Humid** | **Low Temp**  **Low Humid** |
| --- | --- | --- | --- | --- | --- | --- |
|  | AN Mean | -0.73 | -0.35 | -0.45 | -0.45 | -0.90 |
|  | 95% CI | -0.99, -0.47 | -0.60, -0.090 | -0.71, -0.20 | -0.70, -0.18 | -1.2, -0.65 |
|  | C4 Mean | -1.4 | -0.95 | -1.03 | -1.2 | -1.39 |
|  | 95% CI | -1.7, -1.2 | -1.2, -0.69 | -1.29, -0.77 | -1.5, -0.97 | -1.7, -1.1 |
|  | SP Mean | -1.5 | -1.5 | -1.5 | -1.5 | -1.2 |
|  | 95% CI | -1.8, -1.2 | -1.7, -1.2 | -1.8, -1.3 | -1.7, -1.2 | -1.5, -0.96 |
|  | TNT Mean | -0.79 | -0.53 | -0.88 | -0.47 | -0.79 |
|  | 95% CI | -1.05, -0.53 | -0.78, -0.27 | -1.14, -0.62 | -0.72, -0.21 | -1.05, -0.53 |
| 331 |  |  |  |  |  |  |

332	*Table 6: Detection limit comparisons for each odorant in each condition vs. standard.*

#### Conditions High Temp High

**Humid**

#### High Temp Low Humid

**Low Temp High Humid**

#### Low Temp Low Humid

| Odor: | t-ratio | P | t-ratio | P | t-ratio | P | t-ratio | P |
| --- | --- | --- | --- | --- | --- | --- | --- | --- |
| AN | 2.2 | 0.11 | 1.6 | 0.15 | 1.7 | 0.15 | -1.02 | 0.31 |
| C4 | 2.7 | 0.03 | 2.3 | 0.05 | 1.1 | .36 | 0.16 | 0.87 |
| SP | 0.22 | 0.91 | -0.16 | 0.91 | 0.12 | 0.91 | 1.7 | 0.41 |
| TNT | 1.6 | 0.25 | -0.51 | 0.61 | 1.9 | 0.24 | 1.3 | 0.25 |

| 333 |  |
| --- | --- |
| 334 | *Canine subcutaneous temperature and detection limit* |
| 335 | Canine subcutaneous temperature across the session is shown in **Error! Reference** |
| 336 | **source not found.**. Only the first 25 minutes are shown due to the variable session length to |
| 337 | complete reversals. Most data are captured within 25 minute sessions. Similar to that observed |
| 338 | by Fernandez et al. (in review), canine subcutaneous temperatures were highest in the high |
| 339 | temperature and high humidity condition. A simple mixed effect model evaluated the impact of |
| 340 | environmental condition on the temperature recorded (with a random effect of dog). Condition |
| 341 | had a significant impact on subcutaneous temperature (Ꭓ^2^=1082, df=4, p<0.0001). Post hoc tests |
|  | *Figure 3 Canine internal temperature change throughout a session, broken down by condition. The maximum* |
|  | *session time was 40m, however most dogs finished the detection task within 20 min (which is shown). Error bars* |
|  | *show the 95% boot strap estimated confidence intervals.* |
| 342 | indicate subcutaneous temperature significantly varied between all conditions except |
| 343 | comparisons between the two low temperature conditions, which were non-significant (est = - |
| 344 | 0.071, t = -0.75 , p = 0.96). |
| 345 | Subcutaneous temperatures in the two low temperature conditions were not accurate and |
| 346 | as such were dropped from further analysis. The readings the HomeAgain™ Universal |
| 347 | Worldscan™ Reader Plus (by MERCK animal health) in low temperature are physiologically |
| 348 | impossible for non-hypothermic dogs. These errors were likely from the chip being impacted by |
| 349 | the temperature condition in the environmental chamber. These chips were not reading core |
| 350 | temperature, which would have been a more accurate measure of body temperature. |
| 351 | Next, we evaluated the impact of mean subcutaneous temperature within a session on |
| 352 | measured thresholds in the standard and two high temperature conditions. We conducted a mixed |
| 353 | effect model to evaluate whether threshold was predicted by the average subcutaneous |
| 354 | temperature across the first 25 minutes of the session (for the two hot and standard conditions), |
| 355 | odor and their interaction. There was no significant interaction between odor and mean |
| 356 | temperature on threshold (Ꭓ^2^=2.2, df=3, p=0.54). Threshold was however impacted by odor |
| 357 | (Ꭓ^2^=122, df=3, p<0.0001) and mean temperature (Ꭓ^2^=7.7, df=1, p=0.005). As mean |
| 358 | subcutaneous temperature in a session increased by a degree C, threshold increased (became |
| 359 | poorer) on average by 0.21 on the log scale. Thus, dogs with higher overall mean temperatures |
| 360 | during a session appeared to have poorer detection thresholds (see Figure 4). Higher mean |
| 361 | subcutaneous temperature was associated with poorer detection limits. This suggests that canine |
| 362 | subcutaneous temperature may be a simple and useful metric to predict or warn handlers of |
| 363 | poorer detection limits. |

364

365 *Latency and Inter-Olfactometer-Interval*
366 Two additional behavioral measures were examined for their relationship to detection
367 threshold. First latency was measured as the time from the start of the trial until the dog inserted
368 their nose into an odor port. Latency to start a trial did differ by the environmental conditions
369 (Ꭓ^2^=10.2, p=0.04). A post hoc test indicates, however, that the only pairwise difference between
370 conditions was that longer latencies were observed in the low temperature high humidity
371 condition compared to standard (t=2.81, p=0.04). Latency neared statistical significance between
372 high temperature high humidity compared to standard (t=2.64, p=0.069).

*Figure 4: Effect of Subcutaneous temperature on detection limits. Line shows the best fit regression for each odor.*

373 Although there was minimal difference in latency between conditions on average, a
374 mixed effect model was fit in which mean session latency, odor, and their interaction predicted
375 log odor detection threshold. Results indicate there was no interaction between odor and latency
376 (Ꭓ^2^=1.248, p=0.74), but there were main effects of latency (Ꭓ^2^=32.29, p<0.0001) and odor
377 (Ꭓ^2^=169.2, p<0.0001). For every 1s mean increase in latency there was a 0.06 increase in log
378 threshold detection limit (poorer detection; see Figure 5).
379 Secondly, mean inter-box-interval was calculated for each session and trial. The inter-
380 box-interval reflected the time the dogs took between nose pokes, reflecting time to get from one
381 box to the next. The inter-box-interval did not vary in a statistically significant manner between
382 the environmental conditions (Ꭓ^2^=6.57, p=0.16) alone. Interestingly, however, the mean session
383 inter-box-interval was related to overall threshold detection. A mixed effect model predicting
384 threshold by the mean session inter-box-interval, odor, and their interaction, indicated that mean
385 inter-box-interval predicted threshold (Ꭓ^2^=10.89, df=1, p=0.001). Odor also predicted threshold
386 as expected (Ꭓ^2^=153.49, df=3, p<0.0001) but the interaction between inter-box-interval and odor
387 only approached statistical significance (Ꭓ^2^=6.87, p=0.07). An average increase in the inter-
388 box-interval of 1s led to an average increase in log threshold of 0.22, indicating that taking
389 longer to move from one box to the next was associated with poorer thresholds (see Figure 6). 390

|  | *Figure 6 Effect of mean session latency on log detection threshold.*  *Figure 5 Effect of the interval between olfactometer boxes on log detection threshold.* |
| --- | --- |
| 391 |  |
| 392 | *Odor availability and detection limit* |
| 393 | One of the goals of this experiment was to determine the interaction of odor availability |
| 394 | and canine detection limits in adverse environmental conditions. If odor availability is a greater |
| 395 | factor on detection limit than canine physiological response to environmental conditions, it |
| 396 | would be expected that the best canine detection would be seen in higher temperatures and |
| 397 | relative humidity. However, this pattern was not observed in the data. |
| 398 | A linear mixed model was run for the low temperature and high temperature conditions, |
| 399 | excluding standard conditions in which odor availability analysis was not performed. This linear |
| 400 | mixed effects model was run to determine if detection limit was predicted by odor availability |
| 401 | (total VOCs in ppm), condition, and odor. Odor type was found to have a significant effect on |
| 402 | detection limit (Ꭓ^2^=109.4, df=3, p<0.0001). However, condition (Ꭓ^2^=7.72, df=3, p=0.052) and |
| 403 | odor availability (Ꭓ^2^=3.50, df=1, p= 0.061) only approached significance. This analysis indicates |
| 404 | that odor availability is not the main driving factor impacting detection limit, instead the |
| 405 | physiological and behavioral interactions discussed seem to play a more significant role in |
| 406 | detection limit. |
| 407 | *Experiment 1 conclusion* |
| 408 | Overall, the results from this study suggest that decrements in detection performance |
| 409 | appear to be largely impacted by biological or physiological effects on the canine. This is |
| 410 | exemplified by performance decrements occurring with C4 in high temperature conditions, |
| 411 | especially in high temperature and high humidity conditions where odor availability was its |
| 412 | greatest (Huff, et al. in prep), but canine performance still showed a decrement. Decreased search |
| 413 | speed (longer inter-box-interval and search latency) and increased mean subcutaneous |

414 temperature (for the standard and high temperature conditions) were significantly associated with
415 poorer detection limits. These behavioral and physiological measures further support that the
416 reduced detection limits are related to canine factors rather than odor availability.
417 Although the only significant relationship seen in the data between odorant and
418 environmental condition was between C4 and the high temperature conditions, the results for AN
419 are similar, although not statistically significant. One limitation of the current study design was
420 the small sample size (n=8) with few within-subject replications. It could have been possible to
421 increase the power of this experiment if each dog had been tested in each condition more than
422 once. However, as discussed above, we were concerned that this would lead to potential
423 acclimatization.
424 It was not possible to draw conclusions about threshold detection limits and
425 environmental conditions for smokeless powder or TNT. First the dilution range of the
426 olfactometer used was inadequate to provide the range of dilutions necessary to reach threshold
427 for smokeless powder (lowest threshold was still above detection limit), and the highest dilution
428 was too near the detection ability for TNT. We suspect these floor and ceiling effects could have
429 been reduced if an olfactometer with a larger range of dilutions had been available.
430 An area for future study, based on Experiment 1 would be to make some of the
431 environmental conditions more extreme. In particular, the cold conditions, were not as extreme
432 as what many detection dogs may need to operate in. The low temperature conditions of 0°C was
433 chosen based on the physical limits of the environmental chamber. However, this is not an
434 extreme cold by which many detection dogs may need to work. Thus, further research is
435 necessary is subzero conditions.
436 The results from Experiment 1 indicate that canine detection is poorer in the high
437 temperature conditions compared to the standard condition, However, after Experiment 1 it was
438 not evident if this performance decrement could be reduced with specific training. Because the
439 only significant result between odorant detection limit and condition was seen for C4, in
440 Experiment 2, only C4 was used as the target odorant. The high temperature and high humidity
441 condition was selected as the testing and training condition because that condition resulted in
442 significantly poorer detection compared to the standard condition.

# 443 Experiment 2: Acclimatization training under extreme environments and

444 canine sensitivity for an explosive odorant

### 445 Materials and Methods

446 Participants
447 The same 8 dogs (4 Labrador Retrievers, 4 German Short-haired Pointers) that
448 participated in Experiment 1 participated in Experiment 2. The dogs were assigned randomly
449 into one of two conditions (Acclimatization and Control). These groups worked with the target
450 odorant (C4) but were trained under different environmental acclimatization treatment plans.
451 Animal welfare considerations
452 We maintained an identical criterion for early discontinuation of a session (e.g., failure to
453 respond during trials) to that used in Experiment 1.

| 454 | Odorants |
| --- | --- |
| 455 | C4 was selected as the target odorant in Experiment 2 because it was the target odorant |
| 456 | which showed significant detection limit differences between the two high temperature |
| 457 | conditions and the standard condition. All other vials in the olfactometer were empty and acted |
| 458 | as controls, identical to the procedure in Experiment 1. |
| 459 | Data collection equipment |
| 460 | Olfactometers used were identical to those used in Experiment 1. |
| 461 | Environmental conditions |
| 462 | The same environmental chamber was used as in Experiment 1. However, only two of the |
| 463 | environmental conditions were utilized, the standard conditions (22°C and 50% relative |
| 464 | humidity), and high temperature, high humidity (40°C and 70% relative humidity). In both |
| 465 | conditions there was a 3°C and 5% relative humidity window on either side to allow changes |
| 466  467 | caused by the chamber door opening and closing. |
| 468 | *Table 7: Details the environmental conditions in the acclimatization plan by group. All temperatures are in °C.* |
|  | **Day Acclimatization group Control group (n=4)** |
|  | **(n=4) condition condition** |
|  | 1 21° ± 3°; 50% ± 5% 21° ± 3°; 50% ± 5% |
|  | 2 24° ± 3°; 53% ± 5% 21° ± 3°; 50% ± 5% |
|  | 3 28° ± 3°; 56% ± 5% 21° ± 3°; 50% ± 5% |
|  | 4 31° ± 3°; 59% ± 5% 21° ± 3°; 50% ± 5% |
|  | 5 34° ± 3°; 62% ± 5% 21° ± 3°; 50% ± 5% |
|  | 6 38° ± 3°; 65% ± 5% 21° ± 3°; 50% ± 5% |
|  | 7-22 40° ± 3°; 70% ± 5% 21° ± 3°; 50% ± 5% |
|  | (excluding testing days 10 |
|  | and 22) |
| 469 |  |
| 470 | Canine acclimatization training and testing |
| 471 | The eight dogs were randomly split into two groups: control and acclimatization. This |
| 472 | threshold test followed identical procedures to that of Experiment 1 with the exception of |
| 473 | completing four reversals instead of seven. Both groups received equal amounts of threshold |
| 474 | training. |
| 475 | Dogs in the experimental group received 5-minute exposure with play-based exercise |
| 476 | within the environmental condition followed by a threshold detection session. The purpose of |
| 477 | this pre-odor task exercise was to work the dogs while they were fatigued, which Gazit and |
| 478 | Terkel (2003), found decreased detection limits initially, but over time canines showed |
| 479 | acclimatization. Exercise treatment consisted of either a toy toss (Kong™) or food toss (the food |
| 480 | used as their reward during odor training and testing) prior to the odor threshold assessment. The |
| 481 | time limit of 5 minutes was chosen because the dogs were panting after 5 minutes, but still |
| 482 | willing and able to work without laying down. Prior to the start of the experiment, a preference |
| 483 | test was performed to determine which reward the dog would prefer to work for in this initial 5 |
| 484 | minutes of exercise. In this test, the experimenter tossed a toy and a piece of the dog’s normal |

485 kibble out in front of the restrained dog. The experimenter then released the dog and whichever
486 reward the dog engaged with first was recorded. This procedure was repeated five times, and
487 whichever reward the dog chose more was the reward that was used in acclimatization training.
488 Three of the four dogs initially used the Kong™ as their reward. However, after the dogs reached
489 the high temperature and high humidity condition requirements (Day 7) only 1 dog still engaged
490 with the toy. The other two dogs were switched to a food toss, which they continued to engage
491 with the rest of the study. The environmental conditions for the acclimatization group, slowly
492 escalated over the course of six days to reach the high temperature and high humidity conditions
493 (see Table 7).
494 In contrast, the control group received a 5-minute pet session in the environmental
495 chamber (instead of exercise) followed by a threshold detection session. Critically,
496 environmental conditions remained at standard conditions during the training phase for control
497 dogs and control and experimental dogs received identical olfactory training.
498 Canine testing
499 To ensure that the two experimental groups did not have significantly different detection
500 limits for C4 by random chance, on Day 0, the eight dogs were tested (with C4) in the standard
501 temperature and RH condition, without prior exercise. The threshold detection limit baselines in
502 the high temperature and humidity conditions were established from this session.
503 On day 11 (after 10 days of acclimatization training) and on day 22 (after twenty days of
504 acclimatization training) both groups underwent threshold testing. This testing consisted of the
505 same threshold test done during acclimatization, in the absence of the exercise/pet 5-minute prior
506 and completed at the high temperature and high humidity condition (40°C and 70% RH).
507 Data Analysis
508 Similar to Experiment 1 threshold was calculated as the geometric mean of all four
509 reversal points. Odor threshold data was log transformed for all analyses. Threshold limits were
510 imputed through the same calculations as done in Experiment 1. If dogs’ detection exceeded the
511 maximum dilution capabilities, the lowest dilution point was used as a reversal point for the
512 remaining reversal points. If a dog did not complete the threshold assessment due to meeting a
513 welfare criterion, prior to reaching all four reversal points, the remaining reversal points were
514 imputed as 80%. Additionally, we also analyzed the results without imputing missing threshold
515 values and calculated threshold using the available reversal points prior to the end of the session.
516 516
517 The effect of treatment (group) on detection threshold was evaluated using a linear mixed
518 model effect, where threshold was predicted by treatment group with a random effect of dog. A
519 separate model was fit for baseline, the first test (following 10 days acclimation) and the second
520 test (following 20 days acclimation). Additional models were fit to evaluate how treatment group
521 impacted the dependent variables of subcutaneous temperature, inter-box interval and search
522 latency.
523 523

### 524 Results and discussion

525 Figure 7A shows that there were minimal differences in detection limit at Day 0 under
526 standard conditions, when neither group of dogs was exercised or pet prior to testing. In fact,
527 detection limits were slightly poorer for the acclimation group compared to the control. Figure
528 7B, however, shows that acclimation dogs outperformed control dogs under the high temperature
529 and high humidity conditions averaged across Test 1 and Test 2 with 10 and 20 days of
530 acclimatization training. Figure 7C shows that acclimation dogs showed optimal threshold

*Figure 7: Detection limits for each of the two treatment groups on a baseline standard condition. A) initial test day, B) average of Test 1 and 2, C) Test 1, and D) Test 2 at 20 days. This graph used imputed detection values.*

531 performance following just 10 days of acclimatization training, outperforming control dogs. A
532 similar result is seen for Test 2, although with less variability in the control dogs and slightly
533 more variability for the acclimated dogs.
534 A linear mixed effect model predicting detection threshold by treatment across Test 1 and
535 Test 2 indicated that acclimatization training had a trend effect when using the imputed
536 thresholds (Ꭓ^2^=2.9, df=1, p=0.09) and a statistically significant effect when not imputing
537 missing thresholds (Ꭓ^2^=4.2, df=1, p=0.041). Only one of the eight canines was removed from
538 the testing room during a testing day (Test 1) due to welfare concerns. This dog was in the
539 control group.
540 Figure 8 shows the effect of acclimatization training on (control vs. acclimatization)
541 threshold (imputed values) and for the three measures that were previously found to be
542 associated with detection thresholds in Experiment 1 (mean subcutaneous temperature, mean
543 inter box interval, and mean session latency). In each of the following models temperature, mean
544 session latency, and mean inter box interval were compared between the two treatment groups
545 for the two test days (excluding day 0, which was at standard conditions). A linear mixed effect
546 model indicates that mean temperature was not different by treatment across the two test days at
547 high temperature and high humidity (Ꭓ^2^<0.1 df=1, p=0.98). In contrast, there was a significant
548 difference between treatments on the mean inter-box-interval, such that acclimatization group
549 walked on average 1s faster from one box to the next than control dogs during the test days
550 (Ꭓ^2^=9.66, df=1, p=0.002). In addition, latency almost met the criterion for statistical
551 significance, and showed that acclimatization dogs-initiated trials approximately 3s faster than
552 did controls during the high temperature high humidity tests (Ꭓ^2^=3.35, df=1, p=0.07).

*Figure 8: Effect of acclimatization on detection threshold, subcutaneous temperature, inter box interval and latency. Day 0 is the baseline at standard conditions. Test 1 and Test 2 is under High Temperature and High Humidity conditions following 10 and 20 days of acclimatization, respectively. All threshold values were based on imputed calculations.*

553 Interestingly, subcutaneous temperature was not different between the treatment and
554 control group. This suggests that although behavioral performance improved (e.g., inter-box
555 interval) and detection limits improved, this was not likely directly caused by improved
556 physiological responses to heat stress (e.g., subcutaneous temperature).
557 The acclimatization protocol leveraged for this study combined brief exercise with de-
558 sensitization and exposure training to the environmental condition. Although this combined
559 program (exercise + de-sensitization) led to improved performance, it’s not clear if both
560 components are necessary. Further, it is possible that both components have important effects.
561 However, given that the exercise conditioning was minimal (~5 minutes) it is possible that a
562 more extensive fitness training program (13,14) could lead to greater benefits.
563 Overall, results from the acclimatization treatment show that brief exercise and de-
564 sensitization to working in 40° ± 3°; 70% ± 5% mitigated detection threshold decrements for C4
565 in those environmental conditions. Performance improvements were also observed for mean
566 inter-box interval (search speed). Further, performance improvements were observed after 10
567 days of conditioning, and we did not observe any greater performance gains following 20 days of
568 acclimatization training compared to 10. Future research is needed to investigate separate effects
569 of physical conditioning and de-sensitization to the environmental conditions to identify optimal
570 and sufficient combinations to mitigate performance decrements.
571 571

### 572 General discussion

573 The goal of this study was to determine how canine detection limits for four explosive
574 odorants change with varying environmental conditions, considering both canine factors and
575 odor availability (Experiment 1). An acclimatization plan was also developed to assess how
576 exercise and environmental acclimatization effect canine detection limits in adverse
577 environmental conditions (Experiment 2).
578 The results from Experiment 1 indicated that canine detection limits for C4 in the 21°C
579 70% relative humidity condition was 3.5-fold poorer than at standard conditions. These results
580 suggest that canine physiological factors have a greater impact on detection performance than
581 odor availability, because odor availability is higher in this condition compared to standard
582 (Huff, et al., in prep).
583 Canine subcutaneous temperatures were significantly higher in the 40°C 70% relative
584 humidity condition compared to the 40°C 40% relative humidity condition indicating that
585 humidity does play a role in canine subcutaneous temperature (est = 0.31, t = 3.2, p = 0.013).
586 Likely this is because evaporative cooling is less effective in higher humidity, and panting is one
587 of the main ways canines cool (6). Even within high temperature conditions, higher mean
588 subcutaneous temperatures were significantly related to poorer detection limits. These results
589 support the findings of Gazit and Terkel (2003) which found that dogs with a higher panting ratio
590 (here indicated by higher subcutaneous body temperature) had more difficulty detecting
591 explosives. This suggests that methods which reduce the canine internal body temperature could
592 increase detection limits.
593 Previous studies indicate that some cooling methods work more quickly than others, and
594 therefore are likely preferable for EDC teams working a long search (15). Davis et al. (2019)
595 found that cooling via immersion was faster for cooling gastrointestinal temperatures than by
596 either cold mats or fans. This effect was even true if the water used to immerse the dog was not
597 cool. Water immersion reduces the internal body temperature fastest, and thus may reduce the
598 panting to sniffing ratio the quickest and thus allow EDC teams to return to work more quickly.
599 In addition to changes in subcutaneous temperature, canines that showed increased
600 latency to search and increased inter-box-interval, had poorer detection performance in
601 Experiment 1. Search speed and internal temperature therefore may provide a valuable resource
602 for handlers to optimize canine performance.
603 In Experiment 2 canine detection performance increased for C4 in the 40°C 70% relative
604 humidity condition after 10 days of acclimatization training (training in increased temperatures
605 and exercise) compared to non-acclimated dogs. There were no changes in subcutaneous
606 temperature between groups suggesting there were no physiological changes resulting from the 607	acclimatization plan. However, canines in the acclimatization group did have increased search 608	speed (reduced latency and inter-box-interval). It is unclear from Experiment 2 if the exercise or 609	the increased exposure to adverse environmental conditions or both are necessary for increased 610	detection performance, further research is needed to clarify this relationship.

611	One important limitation for this study is the limited sample size. Nonetheless, in

612	Experiments 1 and 2, clear differences in performance were observed. A second limitation was

613	the dilution capabilities of our olfactometer system. This limited the ability to measure

614	differences between groups because thresholds for some odors reached a ceiling or floor. Future 615	work could utilize multi-stage dilution to generate higher and lower dilutions, which would be 616	more sensitive to detecting odor threshold changes.

617

618	Future directions

619	Future work that refines an acclimatization plan would be valuable. Experiment 2

620 indicated that after just 10 days of increased exposure to adverse environmental conditions with 621 five minutes of exercise canines had better detection for C4 than their counterparts who did not 622 work in high temperature and humidity conditions and were not exercised prior to work.

623	However, it is not clear, based on this research if both exercise and increased exposure to adverse

624	conditions are necessary and whether it is necessary to acclimate dogs for 10 days for better

625	detection limits, or if fewer days could produce the same results, and thus reduce training time.

626	Lastly, our analysis was focused on the decrements caused by high temperatures on C4 627	detection limits. However, our prior work in Experiment 1 demonstrated that canines can also 628	show reduced performance in cold temperatures, particularly cold and humid conditions. Thus, 629	additional work is necessary to explore de-sensitization and/or physical conditioning effects on 630	performance decrements in cold conditions.

631	Conclusions

632	Poorer detection limits for C4 was observed in high temperature (high humidity and low 633 humidity) conditions compared to standard conditions. Similar trends were observed with other 634 energetics, but were less clear due to limitations in odor dilutions our olfactometer system

635	generated. Poorest performance for C4 detection occurred under highest temperature and

636	humidity conditions in which there was highest VOC availability as measured through SPME 637	GC-MS, indicating that performance decrement was likely due to canine factors rather than odor 638	availability In Experiment 2, C4 detection limits increased (better detection) in the high

639	temperature high humidity condition following an acclimatization plan compared to control dogs

640	within 10 days. Improvements were associated with behavioral (inter-box interval) changes

641	rather than changes in subcutaneous temperature.

642

### 643 Acknowledgements

644	The authors would like to thank all husbandry staff, and volunteers for their care of the 645	canines during this project. Special thanks to Avery Bramlett for her help on Experiment 2.

646

647	References

648	1. Furton KG, Myers LJ. The scientific foundation and efficacy of the use of canines as chemical 649	detectors for explosives1Invited paper for the special issue of Talanta ‘Methods for

650	Explosive Analysis and Detection’.1. Talanta. 2001 May 10;54(3):487–500.

651	2. Farr BD, Otto CM, Szymczak JE. Expert Perspectives on the Performance of Explosive 652	Detection Canines: Operational Requirements. Animals. 2021 Jul;11(7):1976.

653	3. Reed SE, Bidlack AL, Hurt A, Getz WM. Detection distance and environmental factors in 654	conservation detection dog surveys. The Journal of Wildlife Management. 2011;75(1):243– 655	51.

656	4. Nussear KE, Esque TC, Heaton JS, Cablk ME, Drake KK, Valetin C, et al. Are Wildlife Detector 657	Dogs or People Better at Finding Desert Tortoises (Gopherus Agassizii)? [Internet]. 2008 Feb 658	[cited 2023 Jan 21]. Available from: https://apps.dtic.mil/sti/citations/ADA486646

659	5. Cablk ME, Sagebiel JC, Heaton JS, Valentin C. Olfaction-based Detection Distance: A 660	Quantitative Analysis of How Far Away Dogs Recognize Tortoise Odor and Follow It to 661	Source. Sensors. 2008 Apr;8(4):2208–22.

662	6. Goldberg MB, Langman VA, Richard Taylor C. Panting in dogs: Paths of air flow in response 663	to heat and exercise. Respiration Physiology. 1981 Mar 1;43(3):327–38.

664	7. Gazit I, Terkel J. Explosives detection by sniffer dogs following strenuous physical activity. 665	Applied Animal Behaviour Science. 2003 Apr 19;81(2):149–61.

666	8. Brustkern M, Thompson R, Lawhon S, Good K, Bunker P, Prada-Tiedemann PA, et al. Effect 667	of Rapid Changes in Environmental Conditions on Canine Detection of Methyl Benzoate - 668	ScienceDirect. Applied Animal Behaviour Science [Internet]. 2023 Apr 18 [cited 2023 May

669 16]; Available from:
670 https://[www.sciencedirect.com/science/article/pii/S0168159123000965?casa_token=Oy1X](http://www.sciencedirect.com/science/article/pii/S0168159123000965?casa_token=Oy1X)
671 HnPkIm8AAAAA:bjyt5FG6kAhPsy7FLPf1eLz-
672 qDCzsiernZNavxf6agNNI6mx8G2Zf3ASubqXAlzMsN-5VxXD
673 9. Hoskinson C, McCain S, Allender MC. Comparison of body temperature readings between 674	an implantable microchip and a cloacal probe in lorikeets (Trichoglossus haematodus sp.). 675	Zoo Biology. 2014;33(5):452–4.

676	10. Bates D, Machler M, Bolker B, Walker S. Fitting Linear Mixed-Effects Models Using lme4. 677	Journal of Statistical Software. 2015;67(1):1–48.

678	11. Fox J, Weisberg S. An R Companion to Applied Regression. SAGE Publications; 2018. 608 p.

679	12. Lenth RV, Buerkner P, Herve M, Love J, Miguez F, Riebl H, et al. emmeans: Estimated

680	Marginal Means, aka Least-Squares Means [Internet]. 2022 [cited 2022 Jun 17]. Available

681	from: https://CRAN.R-project.org/package=emmeans

| 682 | 13. Farr BD, Ramos MT, Otto CM. The Penn Vet Working Dog Center Fit to Work Program: A |
| --- | --- |
| 683 | Formalized Method for Assessing and Developing Foundational Canine Physical Fitness. |
| 684 | Frontiers in Veterinary Science [Internet]. 2020 [cited 2022 Sep 27];7. Available from: |
| 685 | https://[www.frontiersin.org/articles/10.3389/fvets.2020.00470](http://www.frontiersin.org/articles/10.3389/fvets.2020.00470) |
| 686 | 14. Stigall AR, Farr BD, Ramos MT, Otto CM. A Formalized Method to Acclimate Dogs to |
| 687 | Voluntary Treadmill Locomotion at Various Speeds and Inclines. Animals. 2022 |
| 688 | Jan;12(5):567. |
| 689 | 15. Davis MS, Marcellin-Little DJ, O’Connor E. Comparison of Postexercise Cooling Methods in |
| 690 | Working Dogs. J Spec Oper Med. 2019 Jan 1;19(1):56–60. |
| 691 |  |

Figure1	[Click here to access/download;Figure;Fig1.tif](https://www.editorialmanager.com/pone/download.aspx?id=34292339&guid=a29740c0-4201-4cc7-8211-96d9e7c32809&scheme=1)

Figure2	[Click here to access/download;Figure;Fig2.tif](https://www.editorialmanager.com/pone/download.aspx?id=34292341&guid=d041c042-353e-45ec-96e6-7868cbde4815&scheme=1)

Figure3	[Click here to access/download;Figure;Fig3.tif](https://www.editorialmanager.com/pone/download.aspx?id=34292347&guid=1f6b54b2-63a1-4a25-9640-3937650d942c&scheme=1)

Figure4	[Click here to access/download;Figure;Fig4.tif](https://www.editorialmanager.com/pone/download.aspx?id=34292342&guid=0de30bdf-48dd-4093-be3f-73c8c62b13fe&scheme=1)

Figure5	[Click here to access/download;Figure;Fig5.tif](https://www.editorialmanager.com/pone/download.aspx?id=34292354&guid=fc3b0216-f307-4f69-85db-857c00d18cc9&scheme=1)

Figure6	[Click here to access/download;Figure;Fig6.tif](https://www.editorialmanager.com/pone/download.aspx?id=34292357&guid=898c29c5-3f28-4dfa-b49d-270a438e4817&scheme=1)

Figure7	[Click here to access/download;Figure;Fig7.tif](https://www.editorialmanager.com/pone/download.aspx?id=34292831&guid=ad2c7555-3214-4d3c-a9be-96dd06fb0600&scheme=1)

Figure8	[Click here to access/download;Figure;Fig8.tif](https://www.editorialmanager.com/pone/download.aspx?id=34292832&guid=efd26bfb-d4a8-4278-b081-2bb1d27d9f3b&scheme=1)

Experiment1_olfactometer_raw_data

[Click here to access/download](https://www.editorialmanager.com/pone/download.aspx?id=34291348&guid=c1a1fa20-fb70-4bdb-9b05-535e79052d15&scheme=1)

[**Supporting Information** AnalysisData_Stg2_olfactometerdata.csv](https://www.editorialmanager.com/pone/download.aspx?id=34291348&guid=c1a1fa20-fb70-4bdb-9b05-535e79052d15&scheme=1)

Experiment1_Volatile_availability

## [Click here to access/download](https://www.editorialmanager.com/pone/download.aspx?id=34291379&guid=1f57e9b4-8c7d-4c15-a0c1-622cb47d1f6c&scheme=1)

[**Supporting Information** VOC_av.csv](https://www.editorialmanager.com/pone/download.aspx?id=34291379&guid=1f57e9b4-8c7d-4c15-a0c1-622cb47d1f6c&scheme=1)

Experiment1_Temperature_data

## [Click here to access/download](https://www.editorialmanager.com/pone/download.aspx?id=34291382&guid=10d69d85-2ca4-41bd-ba14-80d9dd65b4f4&scheme=1)

[**Supporting Information** all_phase2_TemperatureData.csv](https://www.editorialmanager.com/pone/download.aspx?id=34291382&guid=10d69d85-2ca4-41bd-ba14-80d9dd65b4f4&scheme=1)

Experiment1_Temperature_data_wideformat

## [Click here to access/download](https://www.editorialmanager.com/pone/download.aspx?id=34291383&guid=ad1c01ab-2339-46e4-9f15-91b4429af983&scheme=1)

[**Supporting Information** all_phase2_TemperatureData_wide.csv](https://www.editorialmanager.com/pone/download.aspx?id=34291383&guid=ad1c01ab-2339-46e4-9f15-91b4429af983&scheme=1)

Experiment1_Rcode

## [Click here to access/download](https://www.editorialmanager.com/pone/download.aspx?id=34292338&guid=3e5b38e9-c7d7-4541-8db0-08136733269b&scheme=1)

[**Supporting Information** StageIIAnalysis_Plos1.qmd](https://www.editorialmanager.com/pone/download.aspx?id=34292338&guid=3e5b38e9-c7d7-4541-8db0-08136733269b&scheme=1)

Experiment2_olfactometer_raw_data

## [Click here to access/download](https://www.editorialmanager.com/pone/download.aspx?id=34292384&guid=a6cd8b74-23df-4867-a173-fa97c0538a55&scheme=1)

[**Supporting Information** AnalysisData_Stg3_olfactometerdata.csv](https://www.editorialmanager.com/pone/download.aspx?id=34292384&guid=a6cd8b74-23df-4867-a173-fa97c0538a55&scheme=1)

Experiment1_Temperature_data

## [Click here to access/download](https://www.editorialmanager.com/pone/download.aspx?id=34292910&guid=331505d1-4563-49d0-859e-d0943fa74f20&scheme=1)

[**Supporting Information** all_phase3_TemperatureData.csv](https://www.editorialmanager.com/pone/download.aspx?id=34292910&guid=331505d1-4563-49d0-859e-d0943fa74f20&scheme=1)

Experiment1_Temperature_data_wideformat

## [Click here to access/download](https://www.editorialmanager.com/pone/download.aspx?id=34292911&guid=6da44723-660a-42c0-bc8f-31637ee021cf&scheme=1)

[**Supporting Information** all_phase3_TemperatureData_wide.csv](https://www.editorialmanager.com/pone/download.aspx?id=34292911&guid=6da44723-660a-42c0-bc8f-31637ee021cf&scheme=1)

Experiment2_Rcode

## [Click here to access/download](https://www.editorialmanager.com/pone/download.aspx?id=34292912&guid=4bfe16bc-973a-4e52-982d-3f53f943068e&scheme=1)

[**Supporting Information** StageIIIAnalysis_Plos1.qmd](https://www.editorialmanager.com/pone/download.aspx?id=34292912&guid=4bfe16bc-973a-4e52-982d-3f53f943068e&scheme=1)
