# Supplementary material for: Canine detection of explosives under adverse environmental conditions with and without acclimation training
Source: PLoS One. 2024 Feb 21;19(2):e0297538. doi: 10.1371/journal.pone.0297538 (PMC10881013; doi:10.1371/journal.pone.0297538)
Supplement: Attachment — Submitted filename: Kane_rebuttal letter .docx [file pone.0297538.s008.docx]

Kane Plos1 rebuttal letter

**Reviewer #1:**

There is a minor correction needed, please check the highlighted area in the text. However, there are one or two other areas to look for and give a one more reading to make an improvement in writing.

Thank you, we have reviewed the manuscript and have made several revisions to improve the writing. Special attention was paid to the highlighted sections.

**Reviewer #2:**

This manuscript presents some important results related to the detection of explosives by dogs and the effect of ambient temperature/relative humidity on this skill.

However, some information is still required to understand the results.
How long did the dogs remain inside the climatic chamber at each temperature?

Thank you for noting this. This detail has been added, lines 285-289 (experiment 1); 509-512 (experiment 2).

How was the outside temperature at which they were acclimatized? It is important to show this information in both experiments.

This has been added for experiment 1 in lines 233-237. Dogs were not intentionally acclimated in outdoor temperatures during this study; however, they did have outdoor access and each dog was walked 2x per day. For experiment 2 this was addressed in lines 476-479.


Is there a table/figure citation on line 335?

Yes, we have re-inserted figure 3 (included in submission as Fig3.tif).

This symbol Ꭓ2 does not appear in the manuscript.

Thank you for the comment, all have been changed to X^2^

Reviewer #3: Title :

Superb effort has been made by authors.

Thank you!

Make title eye-catchy for the readers. Make it distinct from other research papers in the same field.

I added a (hopefully) catchy first part to the title. Change to: “Hot dogs; not the bomb: Canine detection of explosive odorants in extreme environmental conditions and development of an acclimatization plan to improve sensitivity”

**Abstract**:

Make initial impression to the point. Start from main aim of current investigation.

Thank you for your suggestion, we have changed the line to read “Canines are one of the best biological detectors of energetic materials available; however, canine detection of explosives is impacted by a number of factors, including environmental effects.”

Write a concluding line reflecting future direction.

We have added this final sentence to the abstract: Future work would endeavor to determine what aspect (exercise or environmental conditioning) are most effective in acclimatization for odor detection work.

**Introduction**:

Highlight the canine and odor interactive effects.

Added lines 128-130 discussing potential interactive factors in higher temperatures.

Improve introduction section with latest references.

We have re-reviewed the literature, using search terms “canine”, “odor detection” and “environment” and all of the relevant literature is included in the introduction

**General Discussion:**

This section is deficient in supported studies. Add previous investigations in line with current findings.

If possible add latest references.

Thank you for this note, several studies have been added to our general discussion to help ground our results in current literature.
